# *In Vitro* Exposure of A549 and J774A.1 Cells to SiO_2_ and TiO_2_ Nanoforms and Related Cellular- and Molecular-Level Effects: Application of Proteomics

**DOI:** 10.1021/acs.jproteome.4c00651

**Published:** 2025-03-04

**Authors:** Premkumari Kumarathasan, Nazila Nazemof, Erica Blais, Krishna Priya Syama, Dalibor Breznan, Yasmine Dirieh, Hiroyuki Aoki, Sadhna Phanse, Azam Tayabali, Mohan Babu

**Affiliations:** † Environmental Health Science and Research Bureau, HECSB, Health Canada, Ottawa, Ontario, Canada K1A 0K9; ‡ Faculty of Health Sciences, University of Ottawa, Ottawa, Ontario, Canada K1N 6N5; § Department of Biochemistry, University of Regina, Regina, Saskatchewan, Canada S4S 0A2

**Keywords:** nanoSiO_2_, nanoTiO_2_, *in vitro* toxicity, nanoforms, oxidative stress, proteomics, cellular pathways

## Abstract

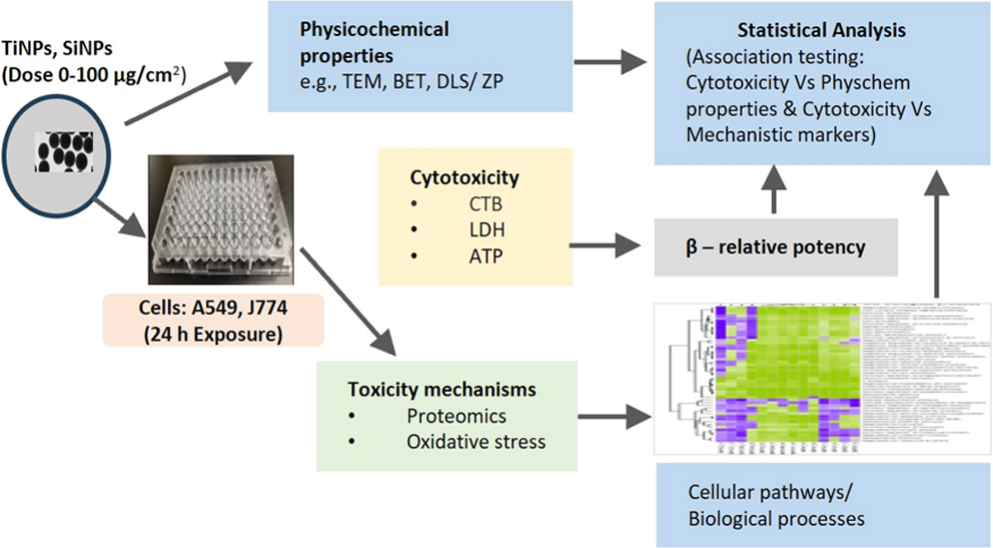

There is an emerging interest in incorporating proteomic data for environmental health risk assessments. Meanwhile, the production and use of engineered nanomaterials (ENMs) with attractive physicochemical properties are expanding with the potential for exposure, thus necessitating toxicity information on these materials for health risk analysis, where proteomic data can be informative. Here, cells (A549 human lung epithelial and J774A.1 mouse monocyte/macrophage cells) were exposed to ENMs (nanoforms of SiO_2_and TiO_2_) of different sizes and surface chemistries (dose: 0–100 μg/cm^2^, 24 h) for *in vitro* toxicity data. Cytotoxicity (CTB, ATP, and LDH), oxidative stress (GSH oxidation), and proteomic analysis (MS- and antibody-based) were conducted post-nanoparticle (NP) exposure to determine the relative potency and identify perturbed cellular pathways. Dose-, nanoform-, and cell type-specific cytotoxicity changes were observed upon exposure to both nanoSiO_2_ and nanoTiO_2_. Size, agglomeration, surface modification, and metal impurities appeared to be the determinants of cytotoxicity. Proteomic analysis identified some enriched mechanistic pathways and biological processes relevant to cell defense/phagocytosis, stress, metabolism, apoptosis, and inflammatory processes in J774A.1 cells exposed to these NPs. A549 cells exhibited enriched pathway/biological processes relevant to transport/endocytosis, stress, metabolism, and inflammatory processes post-NP exposures. Concordance was observed between the nanoform exposure- and cell type-related cytotoxicity responses, notably cellular ATP, which is critical for cell viability, oxidative stress, and cellular pathways/biological processes. These findings demonstrate the application of proteomics in regulatory toxicology and warrant further research in this direction.

## Introduction

Exposure to environmental chemicals ranging from toxic heavy metals to organic pollutants (e.g., plasticizers, per and polyfluoroalkyl substances, and flame retardants) and air pollutants (ambient air particulate matter including nanosized particles) are linked to various adverse health effects.^1−7^ In recent years, engineered nanomaterials (ENMs) that are intentionally designed to have attractive unique physicochemical properties, including smaller sizes (<100 nm), high surface areas, surface modifications, and optical, catalytic, electrical, and magnetic properties have led to their use in various applications including electronics, cosmetics, food packaging/additives, and biomedicine.^8^ Growing interest in ENM applications has led to increased production and use of these materials, enhancing the probability of exposure of humans and the ecosystem to these nanosized materials, thus causing human health concerns.^9,10^ The presence of nanoscale forms of existing substances in commerce has been identified in Canada and is on the Domestic Substances List (DSL) that requires toxicity information for assessment of associated health risks. Among these materials are the inorganic oxide-based ENMs, amorphous nanoSiO_2_, and nanoTiO_2_.^11^

Silica nanoparticles (SiNPs), including mesoporous and amorphous forms, have found widespread use in bioimaging, drug delivery, sensors, semiconductors, cosmetics, food additives, pesticides, and other consumer products.^10,12−17^ Some desired physicochemical properties of SiNPs include large surface area, biocompatibility, potential degradability, adjustability, and mechanical strength.^18^ Based on annual production, SiNPs are ranked second highest in the global market and are among the top 5 widely used ENMs in consumer products.^9,10^ Meanwhile, nanoTiO_2_ particles (TiNPs) are also largely produced ENMs with about 4 million tons in global production and are used in biomedical, electrical, and consumer applications such as photocatalysts, textiles, UV-resistant paints, cosmetics, toothpaste, sunscreens, food additives, and water treatment agents.^19−24^ Since TiO_2_ bulk material is known to be relatively less toxic,^25^ it provides an impetus for nanoTiO_2_ to be considered in various applications. However, emerging evidence suggests that the nanosize of a chemical compound can be relatively more potent than the bulk material, leading to concern regarding exposure to nanoTiO_2_^26,27^ and similar to nanoSiO_2_.

There are several emerging reports on *in vitro* and *in vivo* toxicity testing of nanoSiO_2_ and nanoTiO_2_. Currently, chemical toxicity testing approaches are moving away from *in vivo* exposure models in support of efforts to reduce the use of vertebrate animals for chemical toxicity testing.^28,29^*In vitro* models are evolving as promising alternatives for toxicity screening studies supported by their low costs and high throughput, and enabled by emerging applications of high content of OMIC analyses to yield mechanistic information. Among the toxicity studies reported on nanoSiO_2_, there are relatively more reports on mesoporous forms compared to amorphous nanoSiO_2_.^30^ Also, in general, the focus of many previous studies was to gain information on size-related toxicological effects of nanomaterials, including nanoSiO_2_ and nanoTiO_2_; thus, toxicity data are lacking for different nanoforms of the same chemical that have various applications.^31−36^ Also, most of the *in vitro* exposure studies on nanoSiO_2_ or nanoTiO_2_ have reported on cytotoxicity or genotoxicity findings, and there are relatively few reports delving into toxicity mechanisms, with emerging studies on the use of transcriptomic analysis for this purpose.^37−40^ In addition to the physicochemical properties of these nanomaterials, the high-content OMIC data that provide comprehensive molecular-level information (a new approach methodology, NAM) is of growing interest since it can add value to ENM exposure-related health risk analysis by advancing chemical grouping and read-across processes and by providing information on toxicity mechanisms.^41^ Moreover, as mentioned above, a recent data gap analysis identified amorphous nanoSiO_2_ and nanoTiO_2_ among a number of engineered nanomaterials commercially available in Canada,^11^ which required toxicity information for health risk assessment.

The objective of this study was to obtain information on the relative *in vitro* cytotoxic potencies of well-characterized amorphous nanoSiO_2_ and nanoTiO_2_ (pristine and surface-modified) nanoforms using multiple cell types relevant to the pulmonary toxicity of these nanoparticles (A549 human lung epithelial cells and J774A.1 mouse monocyte/macrophage cells), and the underlying toxicity mechanisms through high-content proteomic analysis. To achieve this, multiple cytotoxicity assays were conducted post-NP exposure to assess cell health, and physicochemical determinants of cytotoxicity were identified by testing for correlations between cytotoxic potencies of NPs and physicochemical properties. The mechanistic basis of toxicity was explored by testing for cellular oxidative stress and analyzing proteomic changes to gain information on cellular pathways/processes affected by exposure to these nanoforms. Reference bulk forms (microscale particles) were also included for cytotoxicity testing. In addition, concordance between cellular cytotoxicity, oxidative stress, and protein markers of inflammatory processes was tested to verify the potential links between key cellular events and cytotoxicity.

## Materials and Methods

### Nanomaterials

#### NanoSiO_2_ (SiNPs)

Custom-synthesized amorphous silicon dioxide nanoparticles (SiNPs), uncoated pristine (15, 30, 50, 75, 100 nm) and surface-modified (–C_3_-COOH, –C_11_-COOH, –NH_2_, and –PEG), were obtained from Advanced Quantum Materials Inc. (AQM, Edmonton, AB, Canada).

#### NanoTiO_2_ (TiNPs)

Ti-01 aeroxide, NIST SRM, anatase 19 nm, and rutile, 37 nm (uncoated); Ti-02 Kronos, surface-treated Al, Si, and Zr (coated); Ti-03 anatase, 50 nm mkNano (uncoated); Ti-04 anatase, 100 nm mkNano (uncoated); Ti-05 rutile, 50 nm mkNano (uncoated); Ti-06 anatase, < 5 nm, mkNano (uncoated); Ti-07 anatase/rutile, 20 nm, silica-coated; Ti-08 anatase/rutile, 20 nm, silica + alumina-coated; Ti-09 anatase/rutile, 20 nm silica + stearic acid-coated; Ti-10 anatase/rutile, 20 nm, silica + silicone oil-coated; and Ti-11 Sigma nanowire (0.1 × 100 μm^2^) were obtained from Dr. L. Johnston of the National Research Council of Canada (Ottawa, ON, Canada). The reference particles included in this work were amorphous nanoSiO_2_ (12 nm, Sigma-Aldrich, ON, Canada) and bulk TiO_2_ (SRM154b, NIST, Gaithersburg, MD) for the *in vitro* cytotoxicity analysis.

### Physicochemical Characteristics

The chemical composition of particles was tested by inductively coupled plasma mass spectrometry and atomic emission spectrometry (ICP-MS/-AES, Varian Vista-Pro, Mulgrave, Australia) on acid-digested (50% HNO_3_, 8 h at 80 °C) filtered samples following previously reported methodologies^42^ (duplicate analyses). The following analyses were performed at the NRC Laboratory (Dr. L. Johnston, NRC, Canada) using previously reported methods:^43,44^ transmission electron microscopy (TEM) was performed using the dispersed particles on Formvar TEM grid (carbon film-covered copper grids), and samples were imaged using an FEI Technai G2 Spirit Twin TEM operated at 120 kV to measure size and shape of the particles; Brunauer–Emmett–Teller (BET) specific surface areas were measured by nitrogen adsorption using the ASAP 2020 System from Micromeritics (GA); the hydrodynamic diameter was measured (triplicate analyses) in liquid media (e.g., DMEM with FBS, water, or ethanol) by dynamic light scattering (DLS), and the surface charge was measured by ζ-potential (ZP) measurements using a Zetasizer Nano ZS (Malvern Instruments, UK); and thermogravimetric analysis (TGA) was performed to assess the surface group coverage using TA Instruments Q500 IR (Waters Limited, ON, Canada).

### Endotoxin Analysis

The particle preparations mentioned above were analyzed for bacterial endotoxins using the chromogenic Limulus Amebocyte Lysate (LAL; Lonza, MD) test, as reported previously.^35^

### Preparation of Nanoparticle Solutions for Dosing Cells

All stock solutions of ENMs (SiNPs and TiNPs) were prepared (3 mg mL^–1^) in deionized water, vortexed for 30 s, and sonicated in a bath sonicator (100 W, 20 kHz, VWR Ultrasonic Cleaner) for 20 min. (Note: All particle solutions used in this study were prepared fresh prior to each exposure.)

### Exposure of Cells to Nanoparticles

Human alveolar type II epithelial (A549) cells and mouse ascites monocyte/macrophage (J774A.1) cells (ATCC, Manassas, VA) were maintained in Dulbecco’s modified Eagle’s medium (DMEM/high glucose) containing phenol red with 10% (v/v) fetal bovine serum (FBS) in a T-75 flask (Corning, NY) and incubated at 37 °C with 5.0% CO_2_.^35^ At 80% confluency, cells were detached and seeded in a 96-well plate at a density of 10,000 cells/well for A549 and 20,000 cells/well for J774A.1 in 100 μL of DMEM (phenol red-free (Hyclone)), with 10% FBS (Hyclone) and incubated for 24 h prior to particle exposure. Stock solutions of particles were diluted with DMEM (phenol red and serum-free) in water, sonicated for an additional 2 min, briefly vortexed, and 100 μL of the diluted particle solutions were added to cell monolayers that already contained 100 μL of DMEM containing 10% FBS to establish particle exposure doses (0–100 μg cm^–2^). The final FBS concentration in the cell culture medium was 5%. After exposure to the particles, the cells were incubated at 37 °C with 5% CO_2_ and 95% relative humidity for 24 h prior to any analysis. All exposure experiments were performed in triplicate with two technical replicates per exposure experiment, and particle exposures were performed under cell-free conditions in parallel to assess any interference with the cytotoxicity assays due to the presence of nanoparticles. Furthermore, the cells, as well as supernatants, were clarified by centrifugation to ensure the removal of any traces of NPs prior to all analyses. Also, cell supernatants and cells treated with 100 μL of PBS containing Halt Protease Inhibitor cocktail (100×) (Thermo Scientific, Nepean, ON, Canada) were stored at −80 °C until further processing for proteomic analyses.

### Cytotoxicity Analysis

Cellular cytotoxicity assays included analyzing the cellular ATP levels for cell metabolism, lactate dehydrogenase (LDH) released for cell membrane integrity, and Cell Titer-Blue (CTB) reduction for cell viability.

Cellular ATP levels were measured using the CellTiter-Glo luminescence assay kit from Promega. After 24 h of exposure to particles, the culture media was removed from the wells, replaced with 100 μL of fresh medium (5% FBS), equilibrated at room temperature (RT) for 30 min, and treated with 100 μL of CellTiter-Glo Reagent. The plate was shaken for 3 min on an orbital shaker to induce cell lysis and incubated at RT for 10 min to stabilize the luminescence signal. The supernatant was transferred to a 96-well V-bottom plate, centrifuged for 10 min at 2000 rpm, and the luminescence signal was read using a POLARstar Omega spectrophotometer plate reader (BMG Lab Tech, Ortenberg, Germany).

To determine the % LDH released, 150 μL aliquots of supernatants from particle-exposed J774A.1 or A549 cells were collected after 24 h, transferred to a 96-well V-shape plate, and centrifuged at 2000 rpm for 10 min. 50 μL of this clarified supernatant was treated with 50 μL of CytoTox-ONE assay reagent (Promega, Madison, WI). The stop solution from this assay kit was added to the reaction mixture after 10 min, and the absorbance was read at 490 nm using a POLARstar Omega spectrophotometer plate reader (BMG Lab Tech, Ortenberg, Germany). Similarly, control cells not treated with particles were lysed 24 h postexposure and analyzed for total LDH content, and these measurements were used to calculate the % LDH released.

Cell viability was assessed based on the premise that metabolically active viable cells can reduce the nonfluorescent dye Resazurin to fluorescent resorufin.^45^ CellTiter-Blue Cell Viability Assay from Promega was used for this purpose. After 24 h of exposure, cell supernatants were removed and replaced with 100 μL of the fresh medium containing 20% CTB reagent (V/V) and incubated for 3 h at 37 °C. Fluorescence measurements were performed (ƛ_Ex_ = 573 and ƛ_Em_ = 600 nm) using a POLARstar Omega spectrophotometer (BMG Lab Tech, Ortenberg, Germany).

### Statistical Analysis

Cytotoxicity data for the particle-exposed groups were normalized to the corresponding dose of zero controls to obtain the fold effect (FE) for each particle dose. A 3-way ANOVA analysis was conducted on SiNP data sets (for both cell types) with size, dose, and mod (modification) as factors. Meanwhile, for TiNPs, a 2-way ANOVA analysis was conducted with treatment and dose as factors on the FE data for cytotoxicity endpoints. Data were transformed (rank-transformed) to meet the conditions of normality and equal variance, as required. Multiple comparison testing was done using Holm–Sidak analysis. Also, potency estimate (β) was derived using FE = (dose + 1)^β^, where β is the rate of change of dose with respect to the logarithm of the fold effect for a given endpoint^46^ and was calculated using CurveExpert v1.4 (D. Hyams, TN). Pearson product-moment correlation analysis was performed to test for the associations between the physicochemical properties of NPs and NP cytotoxic potency β_avg_ values. Additional correlation tests were conducted using either Pearson product-moment correlation or Spearman correlation analysis to determine associations between cytotoxic potency β_avg_ values and cellular GSH/GSSG ratio (oxidative stress: Decrease in GSH/GSSG ratio), as well as cytotoxic potency β_avg_ values and inflammatory cytokines (IL-8 for A549 cells and TNFα for J774A.1 cells). All statistical tests were performed using SigmaPlot version 13.0 (Systat Software, San Jose, CA).

### Mechanistic Analysis

#### Oxidative Stress

For cellular oxidative stress analysis, glutathione (GSH) and oxidized glutathione levels were measured using the GSH/GSSG-Glo assay following the manufacturer’s instructions (Promega, Madison, WI). Briefly, cell supernatants were removed 24 h post-NP exposure (at 30 μg/cm^2^), and 50 μL of the glutathione lysis reagent or oxidized glutathione lysis reagent was added to each well and the plate was shaken at RT for 5 min on a plate shaker. Then, 50 μL of the luciferin generation reagent was added to each well and the plate was shaken gently and incubated at RT for another 30 min, and 100 μL of the luciferin detection reagent was added to each well and shaken briefly. After 15 min, the luminescence signal was measured by using a POLARstar Omega Spectrophotometer Plate Reader (BMG Lab Tech, Ortenberg, Germany).

### Proteomics Analysis

The secreted proteins in the cell culture supernatants were analyzed 24 h post-NP exposure (at a dose of 30 μg/cm^2^) by the affinity-based targeted multiplex protein array methodology using a Milliplex MAP human multiplex panel or a mouse multiplex panel. The analysis was performed using the Bio-Plex Pro multiplex system (Bio-Rad, Hercules, CA) following previously reported procedures.^35,47^

Proteins in cell lysates were processed as follows: the frozen cells containing antiproteases were subjected to three freeze/thaw cycles and were then treated with 1% sodium deoxycholate, vortexed, and sonicated for 10 min (with ice added to the bath), and were centrifuged at 10,000*g* for 10 min. The supernatants were fractionated using molecular weight cutoff filters (MWCO) to obtain fractions of 10–100 kDa, as described previously.^39^ The filtrates obtained from MWCO fractionation were evaporated to complete dryness under a gentle stream of high-purity N_2_ (g). These evaporated fractions were resuspended in 25 μL of 50 mM NH_4_OAc (adjusted to pH 8), treated with 2 μL of ProteaseMax and 10 μL of Promega Trypsin Gold (Promega, Madison, WI), vortexed gently, centrifuged at 5000*g* (1 min), and incubated overnight in a water bath at 37 °C. These samples were treated with Trypsin-Lys C enzyme solution (50 μg/mL) for additional enzymatic digestion, vortexed gently, centrifuged at 5000*g* (1 min), and incubated for 4h in the water bath at 37 °C. The enzymatic digestion reaction was quenched with 5 μL of 5% TFA in deionized water, vortexed (3 s), and centrifuged at 14,000*g* (10 min) to remove any residues. Supernatants were stored at −80 °C for mass spectrometry analysis by LC-Orbitrap MS.

### LC-MS/MS Analysis

Enzymatically digested peptide samples were desalted using ZipTip C_18_ pipette tips (Millipore Sigma, catalog number ZTC18S096, Darmstadt, Germany), according to the manufacturer’s protocol, dried by evaporation using a Speed Vac concentrator (Savant), and stored at −20 °C prior to MS analysis. Before the MS analysis, the dried peptides were immediately resuspended in 0.1% FA. All samples were analyzed using nano-LC coupled to an Orbitrap Exploris mass spectrometer (Thermo Fisher Scientific). Chromatographic separation of the peptides was performed on a Proxeon EASY nLC 1200 System (Thermo Fisher Scientific) equipped with a Thermo Scientific Acclaim PepMap C18 column (15 cm × 50 μm ID, 3 μm, 100 Å), employing a water/ACN/0.1% FA gradient. 5 μL aliquots of reconstituted peptide samples were loaded on an LC column, and the flow rate was set at 0.6 μL/min. Peptides were initially separated with 1% ACN, which was increased to 3% ACN over 2 min, and then increased to 24% ACN over 45 min, followed by a linear increase to 80% ACN over 17 min and a wash period of 10 min with 80% of ACN. The eluted peptides were directly sprayed into a mass spectrometer using positive nanoelectrospray ionization (NSI) at an ion source temperature of 250 °C and an ion spray voltage of 2.1 kV. Full-scan MS spectra (375–1500 *m*/*z*) were acquired using Orbitrap Exploris at 120,000 (*m*/*z* 400) resolution. The automatic gain control settings were 1 × 10^6^ for the full FTMS scans and 5 × 10^4^ for the MS/MS scans. Fragmentation was performed by nanoelectrospray ionization (NSI) in a linear ion trap when the ion intensity was >1500 counts. The most intense ions were isolated for the ion trap NSI with charge states ≥2 and sequentially isolated for fragmentation using the normalized collision energy set at 35%, activation Q at 0.250, and an activation time of 10 ms. The ions selected for MS/MS analysis were dynamically excluded for 30 s. Calibration was performed externally using the Pierce FlexMix Calibration Solution (Thermo Fisher Scientific, catalog number 39239). The Orbitrap Exploris mass spectrometer was operated with Thermo XCalibur software. All RAW files were converted to mzXML using ReAdW-4.3.1.

Database searches of the A549 and J774A.1 SiNP and TiNP mzXML files were primarily carried out using MaxQuant version 1.6.7.0^48^ against human (downloaded July 24, 2020) and mouse (downloaded July 24, 2020) protein sequence databases downloaded from UniProt.^49^ To boost peptide and protein identifications, we also performed searches using two other search algorithms Tide from ms.Crux software suite version 1.0^50^ and MSGF+ ver. 2020.08.05.^51^ Search parameters were set to allow for two missed cleavages, trypsin digestion, and carbamidomethylation of cysteine as a fixed modification, while variable modifications were oxidation of methionine and acetylation of protein N-termini. The peptide search tolerance was set to 4.5 ppm for MS1, and the MS2 fragment tolerance was set to 20 ppm. Both false discovery rates at the peptide-spectrum match and protein levels were set to 0.1, and peptides with a minimum of 7 amino acids were considered for identification. A subsequent 1% false discovery rate by Percolator ver. 3.6^52^ was applied for peptide identification. Mass spectrometry-based proteomics data were deposited to the MassIVE repository under the accession code “MSV000095579”.

All protein fold-change data (normalized to controls) were used to conduct a preranked Gene set enrichment analysis (GSEA) pathway enrichment analysis^53^ to determine pathways that were significantly up- or downregulated in each sample. The normalized enrichment scores (NES) were then hierarchically clustered using Cluster 3.0^54^ and subsequently visualized as a heatmap in Java TreeView.^55^ Furthermore, the fold-change cutoff was set at 1.5, *p* < 0.05, and a *z* score of 2 was used for the identification of canonical pathways and biological functions. Moreover, Gene ontology (GO) analysis was performed to identify biological processes.

## Results

Tables 1 and 2 illustrate the physicochemical properties of the nanoforms of SiO_2_ and TiO_2_ used in this work. TEM sizes in the dry state, BET surface area, DLS size for agglomeration in solution, ζ-potential for surface charge, and the extent of surface coating/functionalization results are from the TGA data. (Note: TEM images of these materials have been reported previously.^42,56^) The tables also provide information on the total and transition metal contents of these samples, as determined from the elemental analysis results. Among the pristine SiNPs, the 15 nm size form had the highest total and transition metal contents compared to the 30 and 75 nm SiNPs (Table 1). The BET surface area was higher for the 15 nm SiNP with surface modification –C_3_COOH, as well as the 30 nm pristine SiNP form, while the DLS size was higher for the 15 nm SiNP with surface modification –NH_2_, and the 30 nm pristine SiNP form compared to the rest of the SiNP forms. SiNPs with –C_3_COOH and –C_11_COOH surface groups displayed higher ZP (ζ-potential) values compared to the other SiNPs. Among the nanoforms of TiNPs, Ti-02 (surface-modified), Ti-04 (pristine), and Ti-11 (nanowire) displayed higher TEM sizes, while the DLS size was the highest for Ti-10 TiNP (Table 2).

**Table 1:** Physicochemical Properties of SiNPs^a^

SiNP ID	TEM size (nm) (SD)	BET SA (m^2^/g)	DLS size (nm)	PDI	ZP (mV)	total metals (ppm)	transition metals (ppm)	TGA functional groups (μmol g^–1^)
15 nm-P	17 (4)	29.6	657	0.54	–15.9	848	785	
30 nm-P	29 (4)	97.2	1128	0.72	–13.4	616	541	
75 nm-P	78 (6)	17	260	0.29	–16.6	476	419	
15 nm-C_3_- COOH		162	531	0.50	–17.8			351
15 nm-C_11_- COOH		20.1	445	0.44	–21.4			325
15 nm-NH_2_		9.2	822	0.72	–6.7			1232
15 nm-PEG	14 (1)	19.6	528	0.47	–2.1			180

a TEM – transmission electron microscopy, SA – surface area, DLS – dynamic light scattering, PDI – polydispersity index, and ZP – ζ-potential.

**Table 2:** Physicochemical Properties of TiNPs^a^

TiNP ID	TEM size (nm)^b^	TEM size (nm)^c^ (SD)	BET SA (m^2^/g)	DLS size (nm)^d^	PDI	ZP (mV)	total Metals (ppm)	transition metals (ppm)	transition metal content (without Ti) (ppm)
Ti-01	28	19 (6)	54.2	154	0.14	40	517,160	517,070	41
Ti-02	350	209 (60)	16.5	296	0.16	42	506,268	486,657	37
Ti-03	50	30 (7)	82.4	200	0.37	–25	461,848	459,441	81
Ti-04	100	31 (10)	90.9	311	0.35	35	602,288	600,074	58
Ti-05	50	26 (8)	24.0	180	0.13	–29	513,808	510,838	161
Ti-06	5		152	31.7	0.15	33	455,888	455,742	90
Ti-07	20	38 (14)	45.5	484	0.36	–27	472,177	469,416	64
Ti-08	20	40 (13)	27.8	402	0.30	–26	479,422	458,413	100
Ti-09	20	36 (11)	40.8	256	0.18	–12	496,283	493,429	63
Ti-10	20	42 (15)	16.3	614	0.22	–40	452,244	446,071	53
Ti-11	100	790 (377)	23.6	513	0.33		478,281	399,787	54
TiO_2_				513					

a TEM – transmission electron microscopy, SA – surface area, DLS – dynamic light scattering, PDI −polydispersity index, and ZP – ζ-potential.

b Manufacturer’s data.

c NRC data.

d Water except for Ti-09 and Ti-10 in ethanol, for DMEM with FBS medium values, see ref^69^. Ti-01 – anatase and rutile (uncoated); Ti-02 – surface-treated Al, Si, and Zr; Ti-03 – anatase (uncoated); Ti-04 – anatase (uncoated); Ti-05 – rutile (uncoated); Ti-06 – anatase (uncoated); Ti-07 – anatase/rutile, silica-coated; Ti-08 – anatase/rutile, silica + alumina-coated; Ti-09 – anatase/rutile, silica + stearic acid-coated; Ti-10 – anatase/rutile, silica + silicone oil-coated; Ti-11–nanowire (0.1 × 100 μm).

The BET surface area values were higher for Ti-03 and Ti-04 TiNPs, while the ZP values were higher for Ti-01, Ti-02 (+ve), and Ti-10 (−ve) compared to other TiNPs. The total metal content was the highest for Ti-04, while the transition metal content (not including Ti content) was the highest for the Ti-05 TiNP form.

The relative *in vitro* potency values (β) for the different SiNP nanoforms assessed in this work for the two cell types based on individual cytotoxicity assays and also the corresponding consensus potencies (β_avg_) are provided in Table 3. Excluding the positive control SiNP 12 nm, the uncoated pristine SiNP particles generally exhibited higher β values for % LDH release and ATP levels for both cell types (Table 3). Also, the SiNP nanoform exposure-related dose–response profiles for the different cytotoxicity endpoints and for the two cell types are depicted in Figures S1a–c and S2a–c. Here, 3-way ANOVA results revealed size- or dose-main effects or size X mod or dose X mod or size X dose interactions for both cell types, based on the assay. Meanwhile, for the TiNP nanoforms, with A549 cells, Ti-11 exhibited the highest β values for ATP and LDH assays compared to the other nanoforms, and with J774A.1 cells, Ti-08, Ti-11, and Ti-04 exhibited relatively higher β values for ATP and LDH assays (Table 4). The corresponding dose–response profiles for the different TiNP nanoform exposures in the two different cell types are shown in Figures S3a–c and S4a–c. The 2-way ANOVA results showed Treatment- and Dose-main effects for both cell types. In this work, endotoxin levels were negligible in these NPs (data not shown).

**Table 3:** Cytotoxic Potency of SiNPs in A549 and J774A.1 Cells

	A549 cells				J774A.1 cells			
	β				β			
NP	LDH	ATP	CTB	β_avg_	LDH	ATP	CTB	β_avg_
SiNP-12	0.065	0.037	0.01	0.037	0.355	0.36	0.263	0.326
SiNP-15P	0.04	0.033	0.015	0.029	0.068	0.027	0.006	0.034
SiNP-15 C3	0.01	0.018	0.018	0.015	0.001	0.012	0.005	0.006
SiNP-15 C11	0.053	0.008	0.008	0.023	0.011	0.024	0.009	0.015
SiNP-15 NH2	0.071	0.012	0.013	0.032	0.016	0.011	0.016	0.014
SiNP-15 PEG	0.002	0.021	0.01	0.011	0.001	0.026	0.001	0.009
SiNP-30 P	0.018	0.018	0.018	0.018	0.05	0.012	0.001	0.021
SiNP-30 C3	0.019	0.009	0.022	0.017	0.005	0.004	0.007	0.005
SiNP-30 C11	0.142	0.016	0.019	0.059	0.027	0.015	0.003	0.015
SiNP-30 NH2	0.083	0.051	0.046	0.06	0.06	0.008	0.012	0.027
SiNP-30 PEG	0.001	0.014	0.011	0.009	0.004	0.0001	0.007	0.004
SiNP-50 P	0.021	0.01	0.013	0.015	0.055	0.002	0.002	0.019
SiNP-50 C3	0.057	0.016	0.005	0.026	0.021	0.019	0.01	0.016
SiNP-50 C11	0.108	0.01	0.006	0.041	0.029	0.004	0.003	0.012
SiNP-50 NH2	0.021	0.024	0.004	0.016	0.027	0.001	0.008	0.012
SiNP-50 PEG	0.007	0.004	0.004	0.005	0	0.018	0.011	0.01
SiNP-75 P	0.019	0.009	0.002	0.01	0.114	0.021	0.022	0.052
SiNP-75 C3	0.009	0.001	0.006	0.005	0.01	0.006	0.012	0.009
SiNP-75 C11	0.02	0.007	0.001	0.01	0.016	0.007	0.018	0.014
SiNP-75 NH2	0.032	0.006	0.005	0.014	0.096	0.02	0.027	0.047
SiNP-75 PEG	0.006	0.006	0.005	0.006	0.002	0.002	0.019	0.008
SiNP-100 P	0.032	0.015	0.001	0.016	0.138	0.019	0.029	0.062
SiNP-100 C3	0.007	0.006	0.009	0.007	0.003	0.009	0.013	0.008
SiNP-100 C11	0.014	0.001	0.008	0.008	0.011	0.005	0.005	0.007
SiNP-100 NH2	0.016	0.005	0.03	0.017	0.026	0.001	0.013	0.013
SiNP-100 PEG	0.0004	0.048	0.007	0.018	0.013	0.015	0.001	0.01

**Table 4:** Cytotoxic Potency of TiNPs in A549 and J774A.1 Cells

	A549 cells				J774A.1 Cells			
	β				β			
NP	LDH	ATP	CTB	β_avg_	LDH	ATP	CTB	β_avg_
Ti-01	0.0413	0.0172	0.0348	0.0311	0.0319	0.0095	0.0117	0.0177
Ti-02	0.0194	0.0075	0.0298	0.0189	0.0051	0.0052	0.0156	0.0086
Ti-03	0.0212	0.0206	0.0327	0.0248	0.0143	0.0131	0.0157	0.0144
Ti-04	0.0294	0.0094	0.0337	0.0242	0.0422	0.0200	0.0107	0.0243
Ti-05	0.0342	0.0051	0.0318	0.0237	0.0074	0.0205	0.0104	0.0128
Ti-06	0.0379	0.0045	0.0228	0.0217	0.0210	0.0163	0.0066	0.0147
Ti-07	0.0331	0.0017	0.0260	0.0203	0.0190	0.0133	0.0135	0.0153
Ti-08	0.0348	0.0057	0.0250	0.0218	0.0424	0.0192	0.0337	0.0318
Ti-09	0.0349	0.0141	0.0114	0.0201	0.0041	0.0177	0.0166	0.0128
Ti-10	0.0396	0.0269	0.0295	0.0320	0.0022	0.0140	0.0183	0.0115
Ti-11	0.0569	0.0348	0.0235	0.0384	0.0546	0.0196	0.0101	0.0281
TiO_2_	0.0380	0.0268	0.0285	0.0311	0.0079	0.0218	0.0033	0.0110

The average cytotoxic potency-based rankings are provided in Tables 5 and 6 for the SiNP and TiNP nanoforms for both A549 and J774A.1 cell exposure. Among the SiNPs, the smaller-sized pristine (uncoated) and coated ones were relatively more potent in A549 cells than in the J774A.1 cells, where the pristine SiNPs, both small and large particles, were relatively more potent. However, with TiNPs, both uncoated Ti-01 and coated Ti-10, and nanowire Ti-11 were relatively more potent compared to other nanoforms in A549 while in J774A.1 cells, uncoated Ti-04, coated Ti-08, and nanowire Ti-11 were more potent than the other nanoforms.

**Table 5:** Relative Cytotoxic Potencies of SiNPs in A549 and J774A.1^a^

particle	SiNP-12	SiNP-15P	SiNP-15 C3	SiNP-15 C11	SiNP-15 NH_2_	SiNP-15 PEG	SiNP-30 P	SiNP-75 P
A549								
β_avg_	0.037	0.029	0.015	0.023	0.032	0.011	0.018	0.01
rank	1	3	6	4	2	7	5	8
J774A.1								
β_avg_	0.326	0.034	0.006	0.015	0.014	0.009	0.021	0.052
rank	1	3	8	5	6	7	4	2

a β_avg._ = β(LDH) + β(ATP) + β(CTB)/3.

**Table 6:** Relative Cytotoxic Potencies of TiNPs in A549 and J774A.1^a^

particle	Ti-01	Ti-02	Ti-03	Ti-04	Ti-05	Ti-06	Ti-07	Ti-08	Ti-09	Ti-10	Ti-11	TiO_2_
A549												
β_avg_	0.031	0.019	0.025	0.024	0.024	0.022	0.02	0.022	0.02	0.032	0.038	0.031
rank	3	8	4	5	5	6	7	6	7	2	1	3
J774A.1												
β_avg_	0.018	0.009	0.014	0.024	0.013	0.015	0.015	0.032	0.013	0.012	0.028	0.011
rank	4	10	6	3	7	5	5	1	7	8	2	9

a β_avg_ = β(LDH) + β(ATP) + β(CTB)/3.

Associations between the physicochemical properties of the SiNP and TiNP nanoforms and consensus average cytotoxic potency estimates “β_avg_” are provided in Tables 7 and 8. SiNP toxic potencies were correlated with TEM, DLS sizes, SA and surface groups for A549 cell exposure, while SiNP potencies were correlated with DLS size, ZP, and surface groups for J774A.1 cell exposure. Meanwhile, for TiNPs, cytotoxic potency was correlated with the metal content in A549 cells, but in the J774A.1 cells, cytotoxic potency was related to the DLS size and metal content. There were borderline associations and trends observed between the NP potency and physicochemical properties (Tables 7 and 8).

**Table 7:** Correlations between Cytotoxic Potencies (β_avg_) of SiNPs and Physicochemical Properties

	β_avg_ for A549 cells vs					β_avg_ for J774A.1 cells vs			
Pearson correlation	TEM size (nm)	DLS size (nm)	SA^a^ (m^2^/g)	agglomeration SA^a^	TGA^b^ (μmol/g)	DLS size^a^ (nm)	DLS size^b^ (nm)	ζ-potential^a^ (mV)	TGA^b^ (μmol/g)
*R*	–0.436	0.438	0.92	0.913	0.505	–0.928	0.661	–0.953	0.479
*p*	0.03	0.029	0.03	0.03	0.023	0.023	0.002	0.012	0.033

a Correlations were conducted only for pristine SiNPs.

b Surface-modified SiNPs.

**Table 8:** Correlations between Cytotoxic Potencies (β_avg_) of TiNPs and Physicochemical Properties

	β_avg_ for A549 cells vs					β_avg_ for J774A.1 cells vs			
Pearson correlation	DLS^a^	DLS^b^	total metal content^b^	total metal content^c^	transition metal^c^ (no Ti)	DLS^a^	TEM size^a^	transition metal^b^ (no Ti)	BET SA^d^
*R*	0.766	0.826	–0.843	0.884	0.989	0.870	0.808	0.969	0.997
*p*	0.075	0.084	0.072	0.046	0.010	0.024	0.052	0.006	0.053

a Uncoated.

b Coated.

c Anatase/rutile (coated and uncoated).

d Anatase (uncoated).

**Figure 1:**
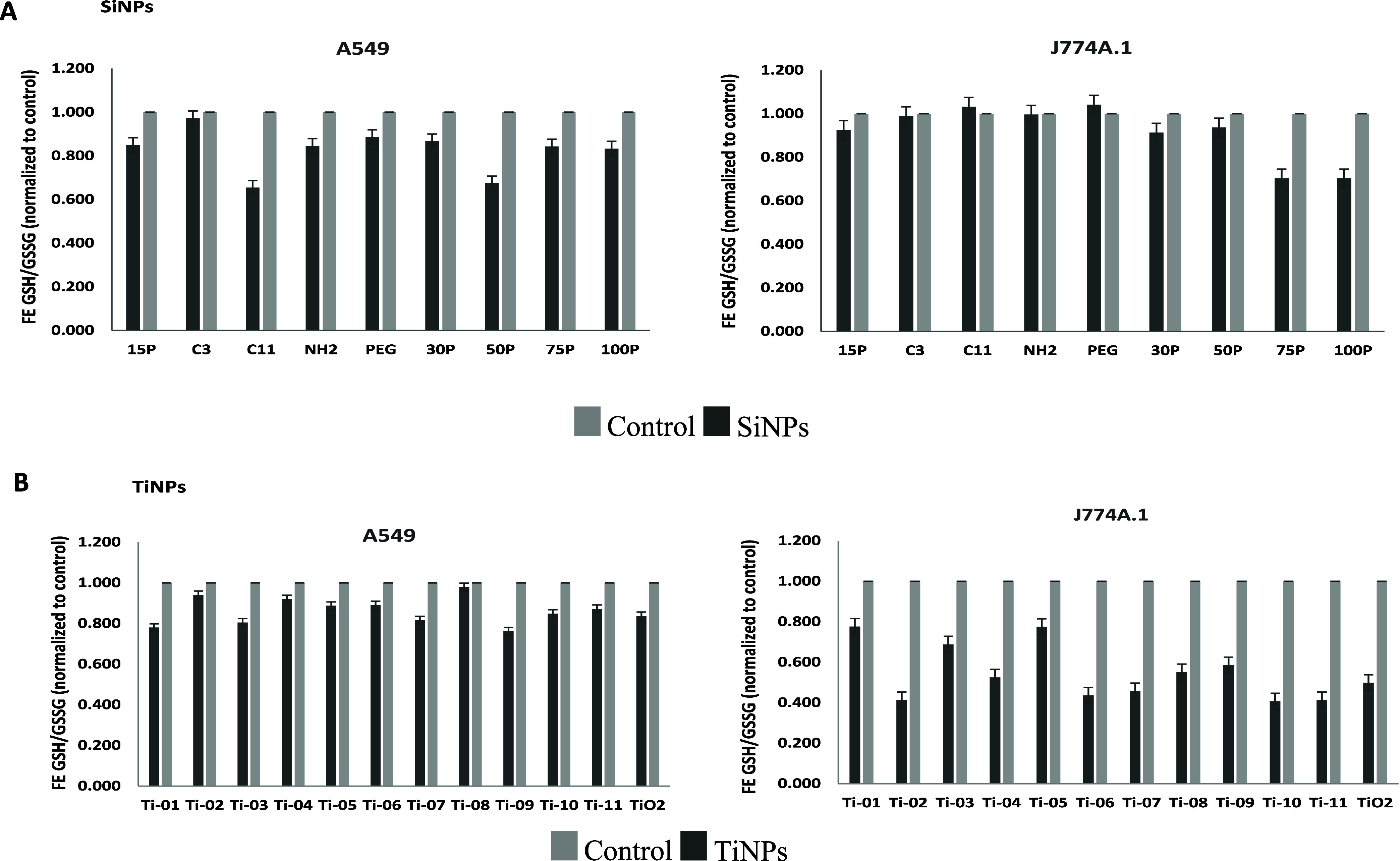
Oxidative stress levels (GSH/GSSG). Cellular oxidative stress measurements are expressed as mean fold change (FE) ± SE (*n* = 3) for A549 and J774A.1 cells exposed to different nanoforms (represented by the black bar) of (A) SiNPs (top panel) and (B) TiNPs (bottom panel), respectively. Cells without any treatment served as the control (represented by the gray bar). Oxidative stress levels in cells are measured by the ratio of GSH (glutathione reduced) to GSSG (glutathione oxidized).

Cellular oxidative stress levels followed by conversion of GSH (reduced form) to GSSG (oxidized form) after exposure of both cell types to the different nanoforms of SiNPs and TiNPs are illustrated in Figure 1A,B. All SiNP nanoforms displayed varying levels of cellular oxidative stress based on the cell type (Figure 1A), with relatively larger uncoated 75 and 100 nm SiNPs exhibiting increased oxidative stress in J774A.1 cells exposed to these particles. TiNP exposure resulted in increased cellular oxidative stress, with contrasting oxidative stress responses after exposure to different nanoforms based on the cell type (Figure 1B).

**Figure 2:**
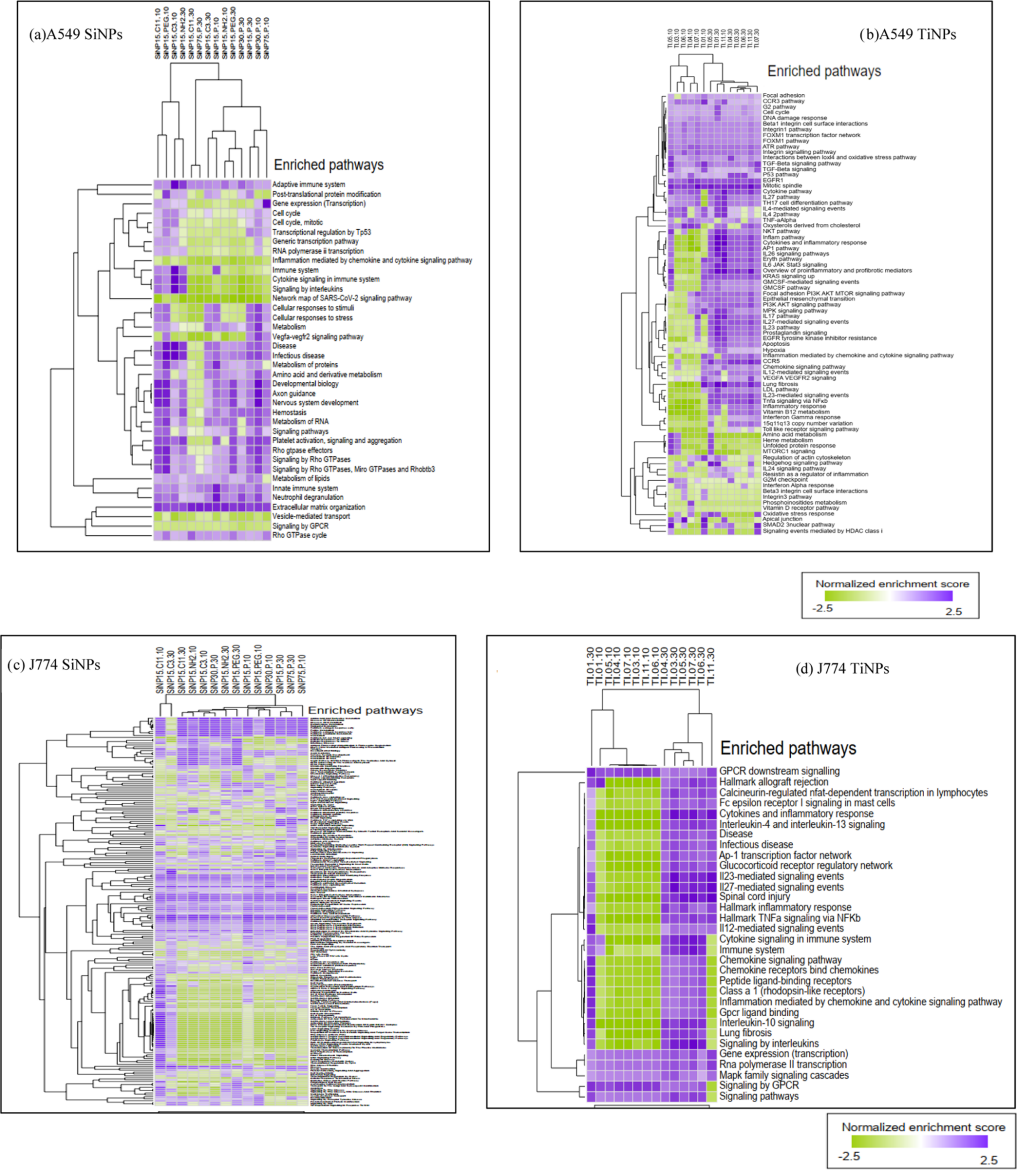
Heat map and hierarchical clustering of enriched cellular pathways (purple – upregulation; green – downregulation) for A549 and J774A.1 cells exposed to (a, c) SiNPs and (b, d) TiNPs from GSEA analysis of proteomic data.

**Figure 3:**
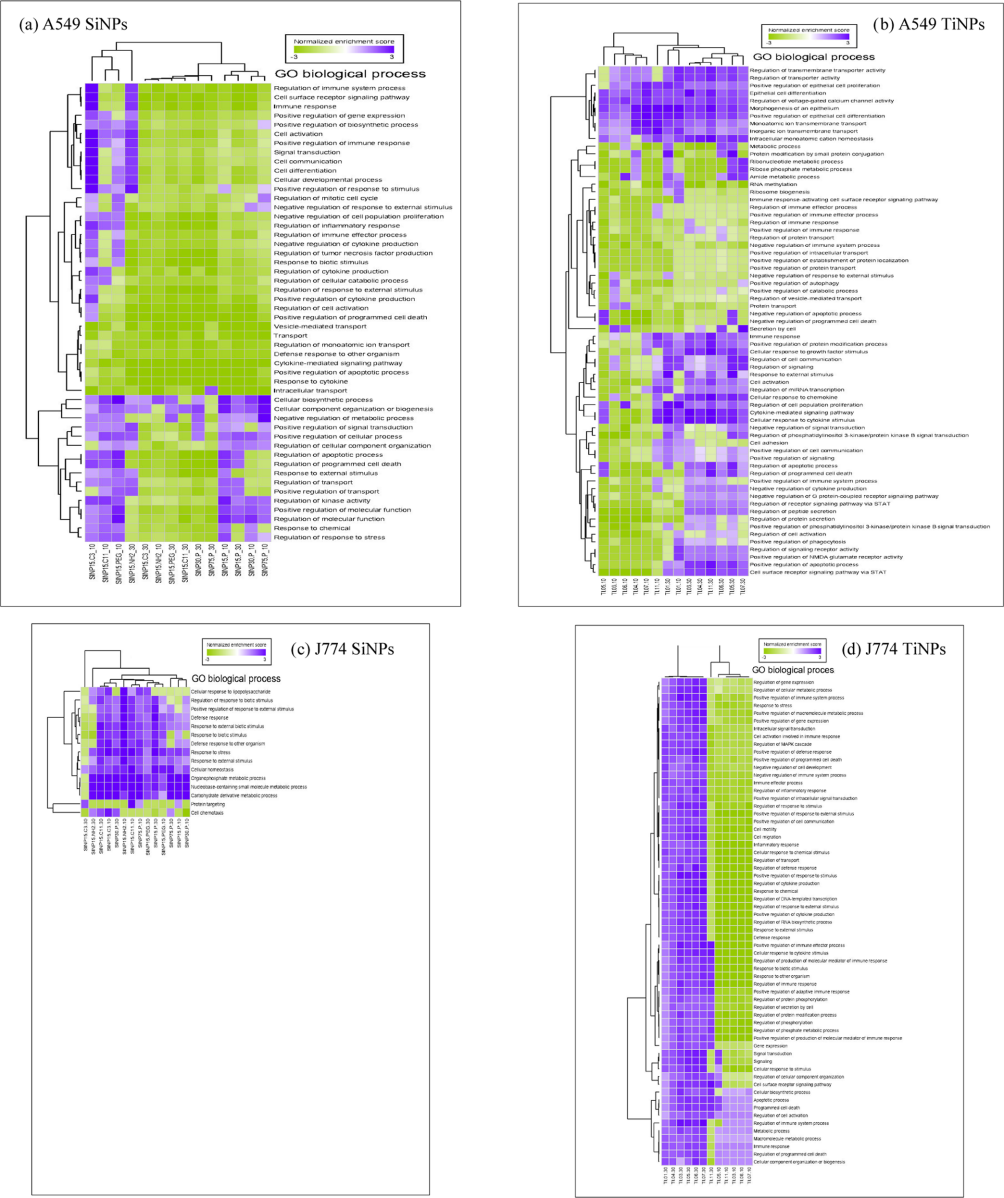
Heat map and hierarchical clustering of biological processes affected (purple – upregulation; green – downregulation) in A549 and J774A.1 cells exposed to (a, c) SiNPs and (b, d) TiNPs.

Gene set enrichment analysis (GSEA) results for enriched cellular pathways and biological processes after exposure of A549 and J774A.1 cells to the different SiNP and TiNP nanoforms are displayed in Figure 2. Green indicates downregulation, and purple indicates upregulation. Dose- and nanoform-specific NP exposure responses were observed across different cell types. In general, the enriched pathways were related to cell signaling associated with various key cellular processes, including particle uptake, inflammatory processes, oxidative stress, cell cycle, metabolism, and cell death, namely apoptosis, as shown in Figure 3.

## Discussion

Although there is extensive evidence supporting the utility of engineered nanomaterials such as nanoSiO_2_ (SiNPs) and nanoTiO_2_ (TiNPs) in various applications, including the biomedical field,^57,58^ clarity is required in terms of the impact of these NP exposures on human health. Among the reports on toxicity testing of these nanomaterials, most studies have focused on size-related effects. Also, in general, toxicity studies on nanomaterials can suffer from the lack of adequate physicochemical characterization of nanomaterials, experimental artifacts arising from assay interferences caused by inherent optical properties of nanomaterials, and contaminants present in the nanoparticles based on routes of synthesis, which can lead to inconsistencies in study findings, making comparisons of findings from different studies difficult. It has been noted that ENM should be considered on a case-by-case basis due to the heterogeneity in their physicochemical properties, which can influence their biological activity.^59−61^ For instance, TiO_2_ nanoparticles in some studies are reported to be less cytotoxic,^62^ while others have reported exposure to these particles by the skin or pulmonary route and showed that these nanoparticles can induce oxidative stress, inflammation,^63^ or genotoxicity.^64^ Systematic studies with well-characterized nanomaterials and defined protocols are needed to obtain a better understanding of the contribution of the physicochemical properties of these nanoparticles, including size, surface groups, solubility characteristics, and contaminants, on their relative toxicities^65−67^ to enable comparison of toxicity findings. In addition, there is limited information on the toxicity of nanoforms of the same chemical, related mechanistic pathways, and physicochemical descriptors of potency required to support the assessment of associated health risks upon exposure to these materials.

Although the routes of exposure to nanoparticles can be inhalation, ingestion and/or dermal, in this work, we examined the *in vitro* toxicities of well-characterized nanoforms of amorphous SiO_2_ (SiNPs) and TiO_2_ (TiNPs) in multiple cell types relevant to the pulmonary toxicity of these particles to understand the effects related to the inhalation toxicity of these materials. Alveolar macrophages and epithelial cells are the gatekeepers of the lung as they are the first line of host defense and have an essential role in maintaining lung homeostasis.^68^ In this context, we chose to use A549 human lung epithelial and J774A.1 mouse monocyte/macrophage cells. Species differences can also allow subsequent corresponding *in vitro*–*in vivo* validation studies as well as provide a comprehensive understanding of these nanoform toxicities. The influence of physicochemical factors on toxic potencies was also assessed in this study, and molecular-level information relevant to cytotoxicity was obtained by the analysis of oxidative stress as well as by application of high-content proteomic analysis. It should be noted here that the solubilities of nanoTiO_2_ particles are low (<1%), as reported earlier,^69^ and amorphous nanoSiO_2_ particles are reported to be relatively slightly soluble (100 mg/L)^70^ in contrast to other highly soluble inorganic oxide nanoparticles such as nanoZnO. Measures were taken in the conduct of exposure experiments in this work to avoid pitfalls in toxicity testing of NMs such as ensuring minimization/tracking of experimental artifacts due to optical interference in cytotoxicity assays by nanoparticles, analyzing the chemical composition of these nanoforms to assess contaminant-related toxicity, and supporting reliable health risk analysis.

Cytotoxicity data on the nanoforms of SiNPs identified size-, dose-specific, size x mod, dose x mod, and size x dose interactions, which were assay-specific and evident in both cell types (Figures S1a–c and S2a–c). Furthermore, cell type-specific changes in cytotoxicity endpoints were clear from the dose–response profiles. For instance, %LDH release, reflecting cell membrane integrity, was adversely affected by smaller-sized (e.g., 15 and 30, nm) particles (uncoated and coated) for A549 cells, while J774A.1 cells were affected by larger particles (e.g., 75 and 100 nm), as shown by the relative potency (β_avg_) values (Tables 3 and 4). In addition, differential cellular ATP levels denoting changes in cell metabolism were observed in both cell types as a result of exposure to the SiNP nanoform (Table 3). Also, since these SiNP nanoforms were custom-synthesized to have a series of sizes with uncoated (pristine) and coated SiNPs, this enabled the assessment of size-, dose-, and modification-related effects on cytotoxicity. Based on the consensus cytotoxic potency (β_avg_) data (Table 3), SiNP particles with contrasting cytotoxic potencies were selected to contain fewer particles, yet to be able to address the effect of size (15, 30, and 75 nm uncoated), and within one size the effect of modification (within 15 nm of different surface modifications) for further testing for mechanistic information such as oxidative stress and proteomic analysis.

The findings on cytotoxicity of TiNPs in the two cell types revealed dose- and treatment-main effects with assay-specific and cell type-specific differences in responses (Figures S3a–c and S4a–c). The TiNP nanoforms used in this work varied in size, surface coating, as well as crystal structure, which can influence the toxicity findings. Although nanoform-specific cellular cytotoxic responses were observed in both cell types, cell type-specific toxicity responses were observed with cellular ATP levels, where most NPs and J774A.1 cells exhibited greater β_ATP_ values and slopes of dose–response relationships compared to those of A549 cells after exposure of these nanoforms (Table 4 and Figures S3a and S4c). Also, a comparison of relative cytotoxic potencies (average potency estimate β averaged values for both cell types) of uncoated 50 nm TiNP nanoforms with different crystal structures (Ti-03 vs Ti-05) showed that the anatase form Ti-03 was relatively more cytotoxic than the rutile form Ti-05, in both cell types (Table 4). These findings are consistent with previous reports, where the anatase structure of nanoTiNP was reported to be relatively more reactive compared to the rutile form and attributed to the larger surface area.^66^ There are also reports relating the crystalline phase and the related increase in the surface area to enhance the ability to generate reactive oxygen species, especially in the case of nanoTiO_2_.^71,72^ In this work, we observed (Table 2) an increased BET surface area and increased DLS size (agglomeration state in solution) for Ti-03 (anatase form) compared to Ti-05 (rutile form). Ti-11, a nanowire, was found to be more potent than the other nanoforms in both cell types. It was also observed that, in general, exposure to relatively larger-size nanoparticles, whether coated or uncoated, affected J774A.1 cells more than A549 cells.

Cell type-specific differences in potency ranking for both SiNP and TiNP nanoform exposure may imply differences in cell–particle interactions and the resulting particle uptake by these cells. Furthermore, correlation analysis results between the physicochemical characteristics and average cytotoxic potencies identified TEM size, DLS size, surface area (SA), and surface groups (TGA results) appeared to be determinants of cytotoxicity for SiNP nanoforms in A549 lung epithelial cells, while DLS size and surface charge indicated by ζ-potential (ZP) and surface groups were identified as descriptors of cytotoxic potency in J774A.1 monocyte/macrophage cells. DLS values reflecting the agglomeration states of custom-synthesized SiNP nanoforms used in this work were in concordance with the agglomeration behavior of commercially available amorphous silica,^35^ where agglomeration state was attributed to the concentration of particles in solution and to the presence of FBS.

For TiNP nanoform exposures, total and transition metal contents appeared to be descriptors of A549 cell cytotoxic potency (significant correlation), with DLS size showing some correlation trends. It has been reported previously that these TiNPs are negligibly soluble,^73^ and solubility may contribute less to the toxicity of these TiNPs. Meanwhile, TEM, DLS size, surface area, and transition metal content appeared to be potent determinants of cytotoxicity for these NP-exposed J774A.1 cells. These findings are in line with previous reports on the association between physicochemical characteristics and toxicity of nanoparticles.^35,43^

For mechanistic analysis, we used exposure doses of 30 μg/cm^2^ and below to avoid examination at doses that caused frank toxicity (high doses where cell death is noticeable) based on cytotoxicity information. Although environmentally relevant doses can be relatively low, these are the typical doses employed in *in vitro* exposure studies to gain mechanistic information. Cellular oxidative stress levels post-SiNP exposure exhibited cell type-related differences. Exposure to all amorphous SiNP nanoforms led to cellular oxidative stress. Also, it was observed that the 15 nm-coated C-11 SiNP nanoform elicited relatively more GSH oxidation in A549 lung epithelial cells (Figure 1A) as opposed to the larger uncoated particles (75 and 100 nm). In J774A.1 cells (Figure 1A), these larger uncoated particles led to increased oxidative stress compared to those of the other nanoforms tested. These cell type-specific observations are in line with the cytotoxic potency β_avg_ values for these nanoforms (Table 3). Furthermore, for SiNP-exposed cells, the cytotoxic potency β_avg_ was negatively correlated with the GSH/GSSG ratio (A549: *r* = −0.24 (not significant); J774A.1: *r* = −0.81 (*p* < 0.01)), suggesting the contribution of oxidative stress to cellular cytotoxicity in both cell types exposed to SiNPs. These findings are consistent with the oxidative potential of amorphous SiNPs.^42,74^ There are also studies that report reactive oxygen species ROS formation as one of the mechanisms by which SiNPs can exert toxicity.^75−80^

Meanwhile, exposure to all TiNP nanoforms led to oxidative stress in both A549 lung epithelial and J774A.1 monocyte/macrophage cells, with the latter showing more GSH transformation. The findings on TiNP exposure-related cellular oxidative stress are consistent with previous reports on the ability of nanoTiO_2_ to participate in electron transfer reactions^81^ and oxidative stress after both *in vitro* and *in vivo* exposure to TiNPs.^82,83^ Similar to the cytotoxicity findings, among the TiNP nanoforms with different crystal structures, the anatase form (Ti-03) displayed increased oxidative capacity compared to the rutile form (Ti-05), for both exposed cell types (Figure 1B), which can be attributed to the relatively increased surface area of the Ti-03 nanoform in contrast to Ti-05, as mentioned before, which is consistent with previous reports.^71,72^ Also, for TiNP exposure, the cytotoxic potency β_avg_ was negatively correlated with the GSH/GSSG ratio (A549: *r* = −0.20 (not sig); J774A.1: *r* = −0.10 (not sig)), suggesting that TiNP exposure-related cellular oxidative stress can contribute to DNA damage, inflammation, and cell injury, which is reflected through changes in cellular pathways and related biological processes (Figures 2 and 3) as well as observed cytotoxicity responses, in line with previous findings.^66,73^

In this work, we analyzed secreted proteins in cell supernatants using targeted antibody-based multiplexed array analysis, while cellular proteins were analyzed by an untargeted LC-orbitrap mass spectrometry method. Also, due to the exploratory nature of this work, we only focused on a (10–100 kDa) fraction of the cellular proteins to reduce complexity and to ensure that there was no interference due to the presence of nanoparticles on protein profiles. Both secreted and cellular protein information were combined for pathway analysis. Pathway enrichment analysis identified some common pathways that were perturbed as a result of cell exposure to nanoforms of SiNPs and TiNPs. Nevertheless, nanoform-, dose- and cell type-specific responses (up/or downregulation) were observed, as illustrated by the heatmaps with hierarchical clustering information (Figure 2). A549 cells exposed to SiO_2_ and TiO_2_ nanoforms exhibited perturbations in mechanistic pathways, including Rho GTPase signaling, cellular response to stress, cytokine signaling, metabolism, cell cycle, and protein post-translational modification. Meanwhile, J774A.1 cells exposed to the nanoforms of SiO_2_ and TiO_2_ also reflected changes in some common pathways, namely, GPCR signaling, MAPK signaling, AP-1 transcription factor network, chemokine, and cytokine signaling pathways. (Figure 2A–D). Although some common pathways were found to be affected by exposure to these nanoparticles, nanoform- as well as dose-specific differences were observed in the pathway profiles (up/down regulation) for nanoSiO_2_ and nanoTiO_2_ for the two different cell types. Also, for SiNP-exposed A549 cells, the cytotoxic potency β_avg_ was positively correlated with secreted IL-8 (*r* = 0.10 (did not reach significance)), and for J774A.1 cells, the cytotoxic potency β was positively correlated with secreted TNFα (*r* = 0.43 (did not reach significance)), where IL-8 and TNFα are proinflammatory cytokines, suggesting some contribution of the inflammatory process to cellular cytotoxicity for both cell types exposed to SiNPs. Similarly, with TiNP exposure, the cytotoxic potency β_avg_ was positively correlated with secreted TNFα (*r* = 0.48, *p* = 0.12) for J774A.1 cells; for A549 cells exposed to uncoated TiNP nanoforms, positive correlation was observed between β_avg_ and secreted IL-8 (*r* = 0.83, *p* = 0.058), suggesting that inflammatory process also contributes to TiNP exposure-related cellular cytotoxicity responses. Meanwhile, the analysis of associations between NP (for both SiNP and TiNP nanoforms) exposure-related cellular GSH/GSSG ratio and cell-released cytokines showed, in general, a negative correlation for both cell types (e.g., SiNP-exposed A549: *r* = −0.67, *p* = 0.058), suggesting a positive association between NP exposure-related oxidative stress and the inflammatory process, implying that oxidative stress may contribute to the inflammatory process, as reported previously for other metal oxide nanoparticles.^66,84^

Moreover, biological processes affected by SiNP and TiNP exposure based on the A549 cellular pathway findings (leading-edge proteins, Table S1) and GO biological process results (Figure 3) were linked to vesicle-mediated transport (clathrin-mediated endocytosis),^85^ oxidative stress, protein post-translational modification, immune/inflammatory processes, DNA damage, oxidative phosphorylation/mitochondria, metabolism, apoptosis/cell death, cell adhesion, and cell cycle. Of these, oxidative and inflammatory processes and their association (correlation analysis data) with cellular cytotoxic potency β support the pathway analysis findings. These mechanistic insights can contribute to the refinement/validation of the adverse outcome pathway (AOP) model, enabling health risk analysis. Additionally, the A549 cells exposed to nanoSiO_2_ showed effects related to scavenger receptor-mediated endocytosis (e.g., STAB2) as well as autophagy, as reported previously,^86,87^ providing some insights into NP–cell interactions. The proteins involved in the pathways affected by SiNP and TiNP exposure were identified to be different for the two particle types in this work, even though they are related to similar biological processes. Furthermore, it was interesting to note that A549 cells exposed to some SiNP nanoforms at high doses showed downregulated pathways related to selected immune/inflammatory processes, while, high-dose exposure to TiNP led to mostly upregulated pathways compared to that at low doses, with the exception of the TiNP nanoform Ti-11, which is a nanowire that displayed downregulation in specific immune/inflammatory pathways as well as pathways relevant to oxidative stress, at both low and high doses of exposure. Low- and high-dose exposure-related protein changes may be due to differences in solubility properties or the shapes of nanoforms. For instance, in the case of Ti-11, the shape may perhaps influence NP–cell interaction modes and subsequent related protein responses, consistent with relatively higher cellular cytotoxic potency (β_avg_). Similar patterns of dampening of oxidative stress as well as immune/inflammatory processes have been observed previously with air particle exposure.^88^ Biological process related to J774A.1 cellular pathway impacted by SiNP and TiNP exposure included defense mechanisms (consistent with the functionality of this phagocytic host–defense cell type), oxidative stress, protein post-translational modification, immune/inflammatory processes, metabolism, and apoptosis/cell death (leading-edge proteins, Table S1 and Figure 3). These findings are consistent with previous reports on cellular pathways/biological processes affected by SiNPs and TiNPs, as identified by various *in vitro* and *in vivo* exposure studies.^35,39,40,43,89^ Dose- and nanoform-specific influences on these pathways are in line with the observed cell type-specific cytotoxicity and oxidative stress responses.

In our previous work on J774A.1 cells exposed to some nanoforms of SiNPs, we have shown localization of nanoparticles in cytoplasmic vesicles, in the vicinity of organelles, including nucleus, mitochondria, and endoplasmic reticulum by TEM analysis, as well as large vacuoles, autophagosomes, loss of cristae in mitochondria and vacuolated mitochondria,^39^ which is consistent with the findings of this study. Both *in vitro* and *in vivo* exposure to SiNP or TiNP and cellular/organ-level toxicity (e.g., respiratory, immune, and reproductive systems) and mitochondrial dysfunction have been reported earlier.^83,90−96^ We have also recently exposed zebrafish embryos to members of the SiNPs and TiNPs tested in this work as an alternative *in vivo* toxicity testing platform and observed NP exposure-related oxidative stress response in the embryos by immunofluorescence (DCFH-DA) and observed protein changes (decrease in superoxide dismutase-SOD2 and increase in barrier-to-autointegration factor) related to heightened oxidative stress after NP exposures (preliminary unpublished information). Also, mitochondrial function-related protein changes (decreased ATP synthase and cytochrome c oxidase subunits) were observed in the embryo tissues (data not shown), implying adverse mitochondrial effects after NP exposure, which is in line with decreased cellular ATP levels (β_ATP_) as well as the observed proteomic changes after *in vitro* NP exposure of the two cell types, in the current study.

*The in vitro* or *in vivo* toxicity of environmental chemicals, including ENMs, has been traditionally examined using one or few cytotoxicity or systemic or physiological endpoints, for risk characterization related to these exposures. Currently, there is a shift toward the application of high-throughput *in vitro* exposure models in combination with high-content molecular-level analysis (e.g., Omics) as new approach methodologies (NAMs), in an effort to move away from the use of vertebrate animals in toxicity testing. There are relatively more reports on transcriptomic and metabolomic analysis for chemical toxicity testing, and lately, proteomic approaches are being considered for this purpose.^15,97−99^ Proteomic screening for chemical toxicity-related molecular-level information can advance the validation and refinement of conceptual models (e.g., adverse outcome pathway (AOP)) used in health risk analysis. Besides, proteomic information, either independently or integrated with other OMIC data (multi-Omic approaches), can enable exposure-specific biomarker development for rapid/cost-effective high-volume sample analysis. Although the proteomic analysis in this work was exploratory and not exhaustive, it provided new insights into key biological events of toxicity (e.g., oxidative stress, inflammatory process, cell cycle, and metabolism/mitochondrial effects) associated with SiNP and TiNP nanoform exposures in multiple cell types, consistent with the observed cytotoxicity responses. The findings from this work on *in vitro* NP exposure-related alterations in inflammatory pathways are in line with recent work on the first human-controlled acute exposure of healthy volunteers to nanoparticles (graphene oxide), suggesting some perturbations in the inflammatory process identified by toxicity testing using plasma proteomics and lipidomics,^100^ irrespective of the nanoparticle type used in the latter work. The current *in vitro* study findings on relative cytotoxic potencies, potential underlying mechanisms, and physicochemical determinants of toxicity not only advance health risk analysis of these SiNP and TiNP nanoforms but also can potentially enable the selection of less toxic nanoforms for different applications.

## Conclusions

Nanoforms of both SiO_2_ and TiO_2_ exhibited dose-, cell type-, and NP-specific cellular cytotoxicity. Size, agglomeration, surface modification, and metal impurities appeared to be some of the important cytotoxicity determinants. Besides, the crystal structure and shape also influenced the cytotoxicity due to TiNP exposure. Cellular pathways affected by SiO_2_ and TiO_2_ nanoforms identified some endocytosis-related processes for A549 epithelial cells, while host defense-related mechanisms were observed in J774A.1 cells, suggesting cell-specific differences relevant to particle uptake mechanisms. Other biological processes affected by exposure to SiNP and TiNP nanoforms in these cell types included oxidative stress, inflammatory processes, cell cycle-related effects, metabolism, and apoptosis/cell death. In addition, the correlation between cellular oxidative stress, inflammatory pathway, and cytotoxic potency adds value in terms of revealing the molecular basis of the toxicity of these nanoforms, which is valuable information for human health risk analysis. These findings warrant future work on high-content proteomic applications in nanotoxicology to unravel the mechanistic pathways underlying the toxicity of these manufactured nanomaterials.

## Supporting Information Available

NP exposure-related biological process, attributed to leading-edge proteins identified by pathway enrichment analysis (Table S1); cytotoxicity analysis in A549 cells exposed to different nanoforms of SiNPs, and dose and surface modification, and size-specific cellular cytotoxicity responses of A549 cells exposed to different nanoforms of SiNPs (Figure S1); cytotoxicity analysis in J774A.1 cells exposed to different nanoforms of SiNPs, and dose-, surface modification, and size-specific cellular cytotoxicity responses of J774A.1 cells exposed to different nanoforms of SiNPs (Figure S2); cytotoxicity analysis in A549 cells exposed to different nanoforms of TiNPs, and dose- and treatment-specific cellular cytotoxicity responses of A549 cells exposed to different nanoforms of TiNPs (Figure S3); cytotoxicity analysis in J774A.1 cells exposed to different nanoforms of TiNPs, and dose- and treatment-specific cellular cytotoxicity responses of J774A.1 cells exposed to different nanoforms of TiNPs (Figure S4). (PDF)
