# Supplementary material for: In Vitro Exposure of A549 and J774A.1 Cells to SiO2 and TiO2 Nanoforms and Related Cellular- and Molecular-Level Effects: Application of Proteomics
Source: J Proteome Res. 2025 Mar 4;24(4):1672–87. doi: 10.1021/acs.jproteome.4c00651 (PMC11976856; doi:10.1021/acs.jproteome.4c00651)
Supplement: Supplementary file 1 — pr4c00651_si_001.pdf [file pr4c00651_si_001.pdf]

Supplementary Information

In Vitro Exposures of A549, J774A.1 Cells to SiO_2, TiO_2 Nanoforms and Related Cellular and Molecular Level Effects: Application of Proteomics

Prem Kumarathasan[1,2*], Nazila Nazemof[1,2], Erica Blais[1], Krishna Priya Syama[1], Dalibor Breznan[1], Yasmine Dirieh[1], Hiroyuki Aoki[3], Sadhna Phanse[3], Azam Tayabali[1], Mohan Babu[3]

[1]Environmental Health Science and Research Bureau, HECSB, Health Canada, Ottawa, ON, Canada K1A0K9

[2]Faculty of Health Sciences, University of Ottawa, Ottawa, ON, Canada K1N 6N5

[3]Department of Biochemistry, University of Regina, Regina, SK, Canada S4S 0A2

***Correspondence:**

Premkumari Kumarathasan, Ph.D

Rm. 330B, 251 Sir Frederick Banting Driveway,

Tunney's Pasture, Ottawa, ON K1A 0K9, Canada.

Phone: 613-218-4530. Email: premkumari.kumarathasan@hc-sc.gc.ca

Table of contents

Supplementary Table S1: NP exposure-related biological process attributed to leading edge proteins identified by pathway enrichment analysis.

Figure S1. Cytotoxicity analysis in A549 cells exposed to different nanoforms of SiNPs.

Dose-, Surface modification and Size-specific cellular cytotoxicity responses ((a) LDH release, (b) ATP levels and (c) CTB reduction) of A549 cells exposed to different nanoforms of SiNPs. Triplicate exposure experiments.

Figure S2. Cytotoxicity analysis in J774A.1 cells exposed to different nanoforms of SiNPs.

Dose-, Surface modification and Size-specific cellular cytotoxicity responses ((a) LDH release, (b) ATP levels and (c) CTB reduction) of J774A.1 cells exposed to different nanoforms of SiNPs. Triplicate exposure experiments.

Figure S3. Cytotoxicity analysis in A549 cells exposed to different nanoforms of TiNPs.

Dose-, Treatment-specific cellular cytotoxicity responses ((a) ATP levels, (b) CTB reduction and (c) LDH release) of A549 cells exposed to different nanoforms of TiNPs. Triplicate exposure experiments.

Figure S4. Cytotoxicity analysis in J774A.1 cells exposed to different nanoforms of TiNPs.

Dose-, Treatment-specific cellular cytotoxicity responses ((a) LDH release, (b) CTB reduction (c) ATP levels) of J774A.1 cells exposed to different nanoforms of TiNPs. Triplicate exposure experiments.

Table S1: NP exposure-related biological process attributed to leading edge proteins identified by pathway enrichment analysis.

	Leading edge proteins in			
	A549 cells exposed to		J774A.1 cells exposed to	
**Biological processes**	**SiNPs**	**TiNPs**	**SiNPs**	**TiNPs**
Oxidative stress	GSTP1, IFNγ, TXNRD1	TxNRD2, XDH	Txn, Txnrdi, Szt2, TNF, Prdx1	TNF-α, IFNγ
DNA Damage	CDK7, PARK7	BAZ1B	-	-
Immune /inflammatory response	CCl2, CFIS, IL10, IFNγ, PARK7	IL6, IL17, TRIM25, CSF2, CCL25	Ywhab	IL4, IL5, IL6, CXCL1, IL2, IL17, CSG, CCL25, TNF-α, IFNγ
Apoptosis / Cell death	GSTP1, PPIA	ARHGAP10, SKIL, HTRA1	SFn, Ywhaz, IL1a, CalrTx, Ctsb	IL1A, Rag1
Metabolism	AKRIBIO, PIP5K1C, PSMD2, GSTP1	DHCR7, ACOX2	PId2, Prkab2	Dgke
Oxidative phosphorylation/ Mitochondria	TXNRDI	-	-	-
Clathrin-, scavenger receptor-mediated endocytosis	PIP5K1C, STAB2, RINL	BMP2, ARHGAP27	-	-
Protein biosynthesis/translation	-	-	RPL4	Eif4g3
Protein post-translational modification(e.g., sulfation, phosphorylation, ubiquitination)	POMGNT2, TRIP	PAPSS2, ST8S1A4	Ywhaz, Papss2, Cbl	Rag1
Cell adhesion	COL6A3, PIP5K1C, CDHI	THY1, TYR03, LAMA4	Ptk2, TjpII, Col4a3	-
Cell cycle	CDK7, SKA2	GAS1	Actb, Calr, Txnrp	
Phagocytosis	-	-	Ptk2, Calr	TNF-α, IFNγ
Antigen presentation/processing	-	-	Psme1	-
Autophagy	PARK7	-	Calr, Depdc5, Prkn	-

(a)

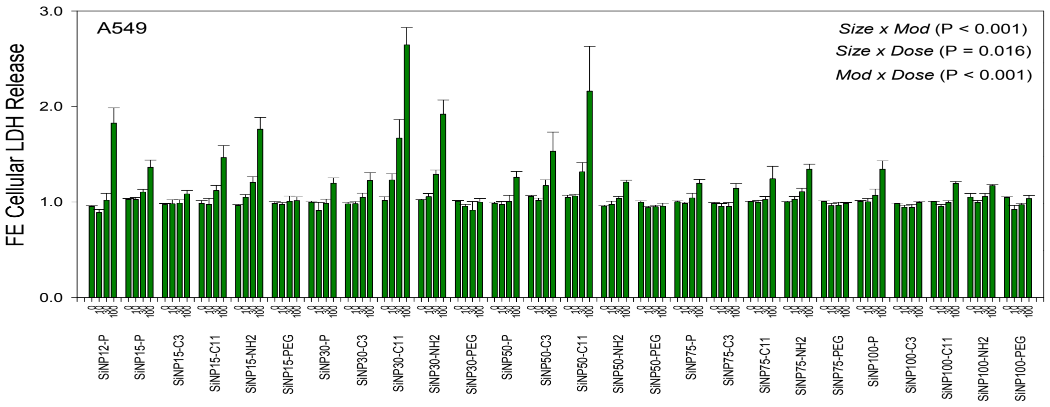

(b)

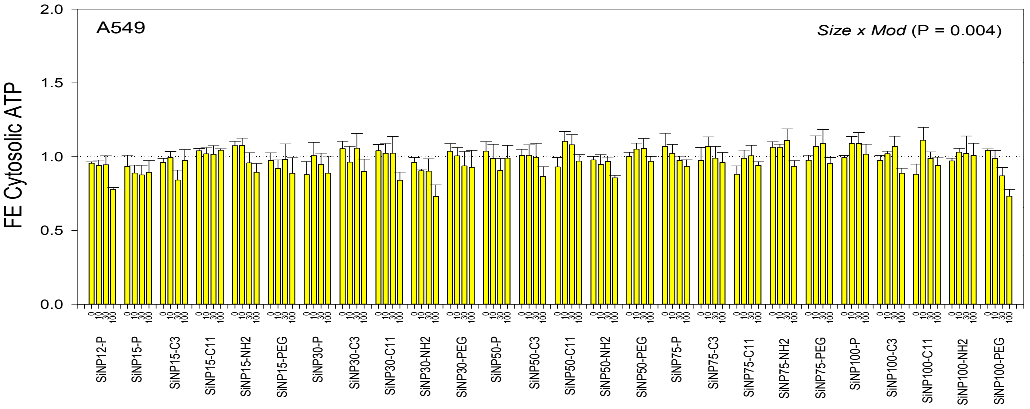

(c)

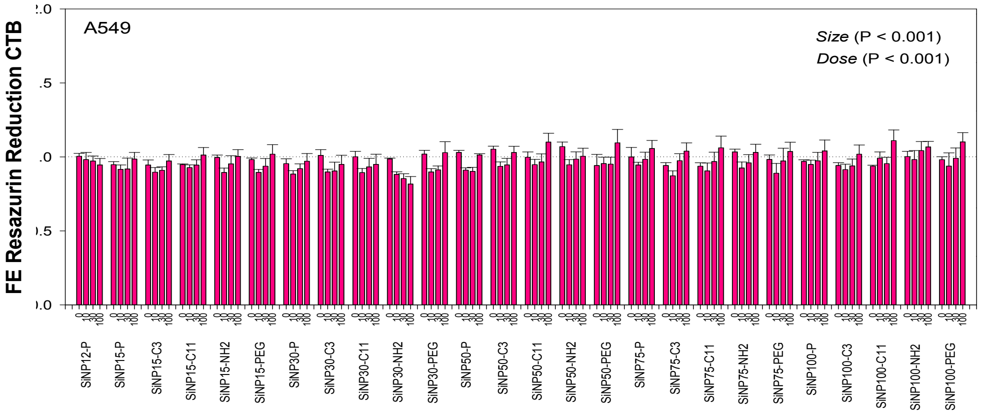

Figure S1. Cytotoxicity analysis in A549 cells exposed to different nanoforms of SiNPs.

Dose-, Surface modification and Size-specific cellular cytotoxicity responses ((a) LDH release, (b) ATP levels and (c) CTB reduction) of A549 cells exposed to different nanoforms of SiNPs. Triplicate exposure experiments.

(a)

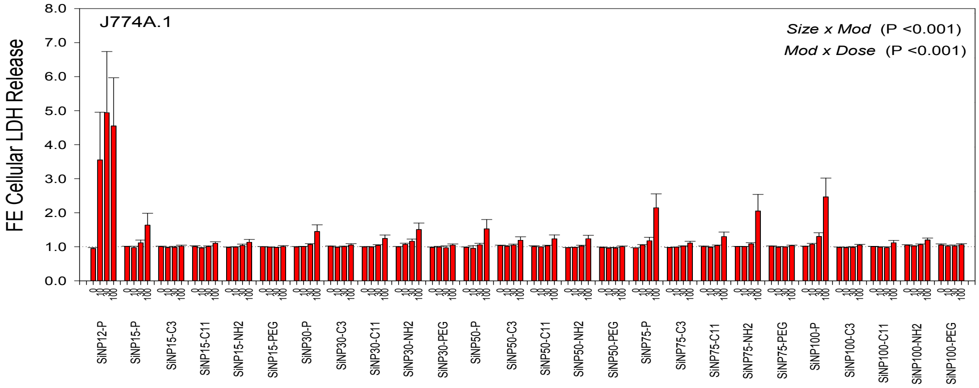

(b)

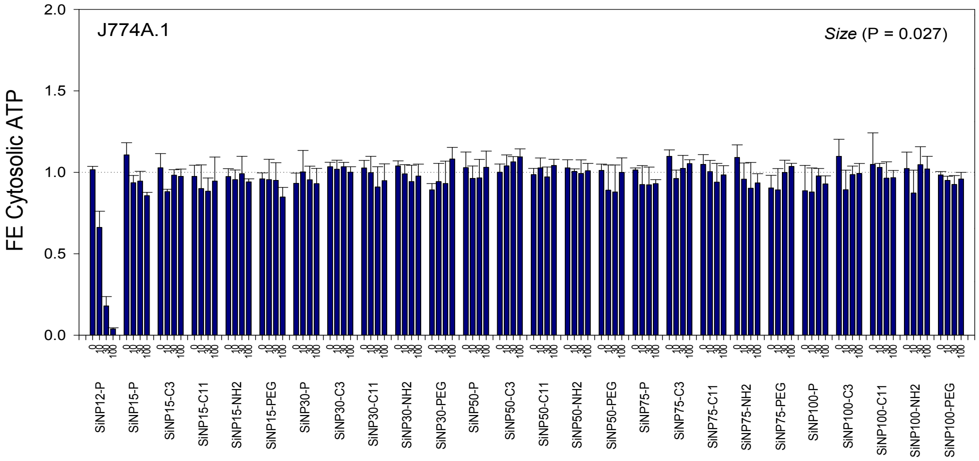

(c)

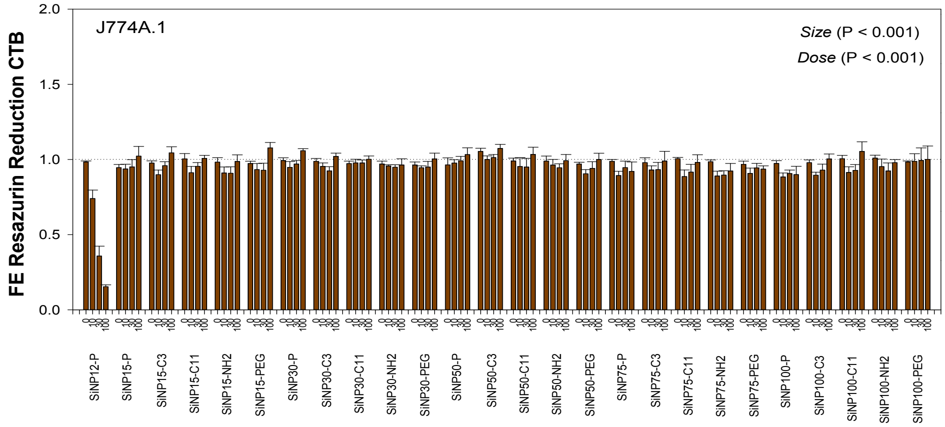

Figure S2. Cytotoxicity analysis in J774A.1 cells exposed to different nanoforms of SiNPs.

Dose-, Surface modification and Size-specific cellular cytotoxicity responses ((a) LDH release, (b) ATP levels and (c) CTB reduction) of J774A.1 cells exposed to different nanoforms of SiNPs. Triplicate exposure experiments.

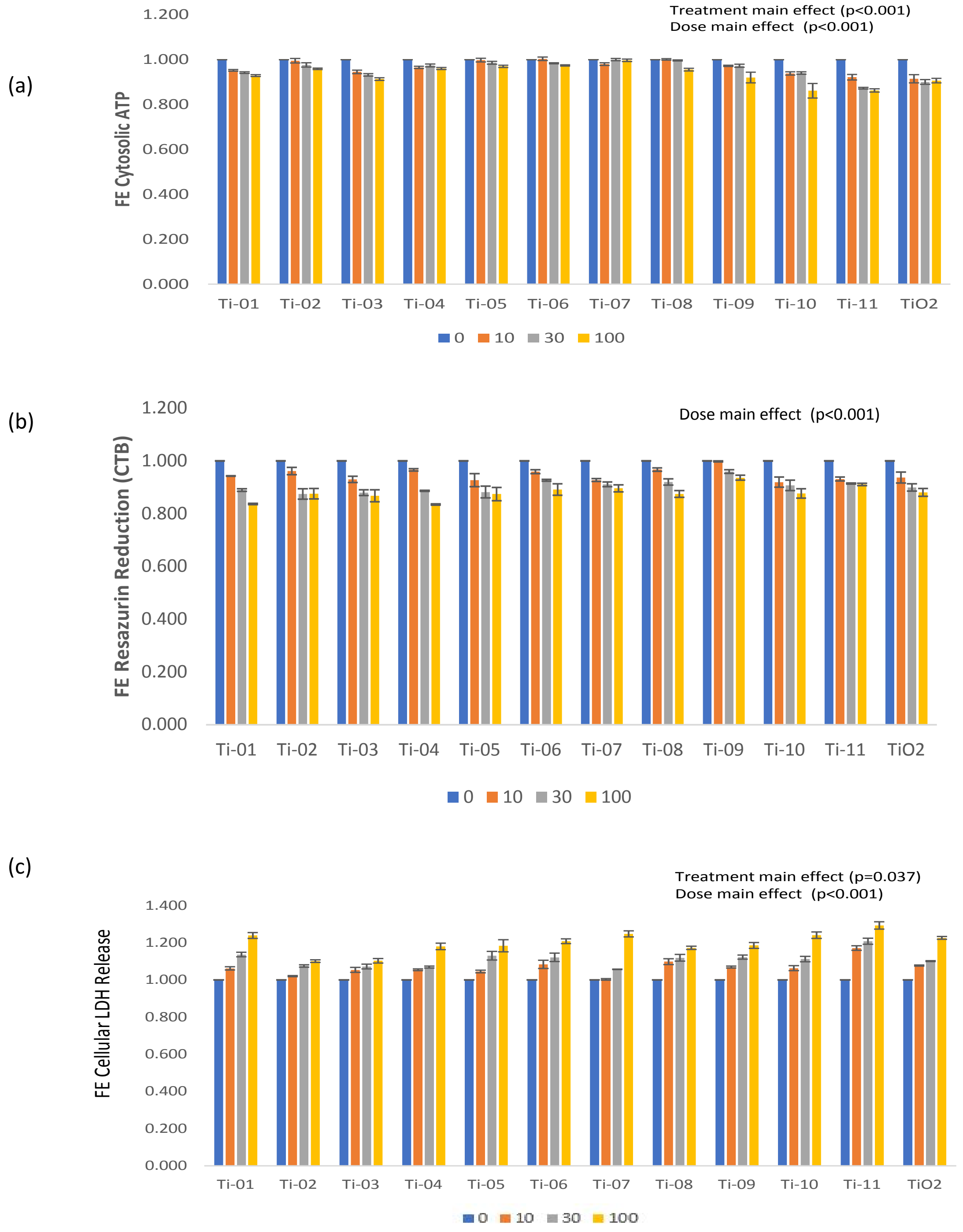

Figure S3. Cytotoxicity analysis in A549 cells exposed to different nanoforms of TiNPs.

Dose-, Treatment-specific cellular cytotoxicity responses ((a) ATP levels, (b) CTB reduction and (c) LDH release) of A549 cells exposed to different nanoforms of TiNPs. Triplicate exposure experiments.

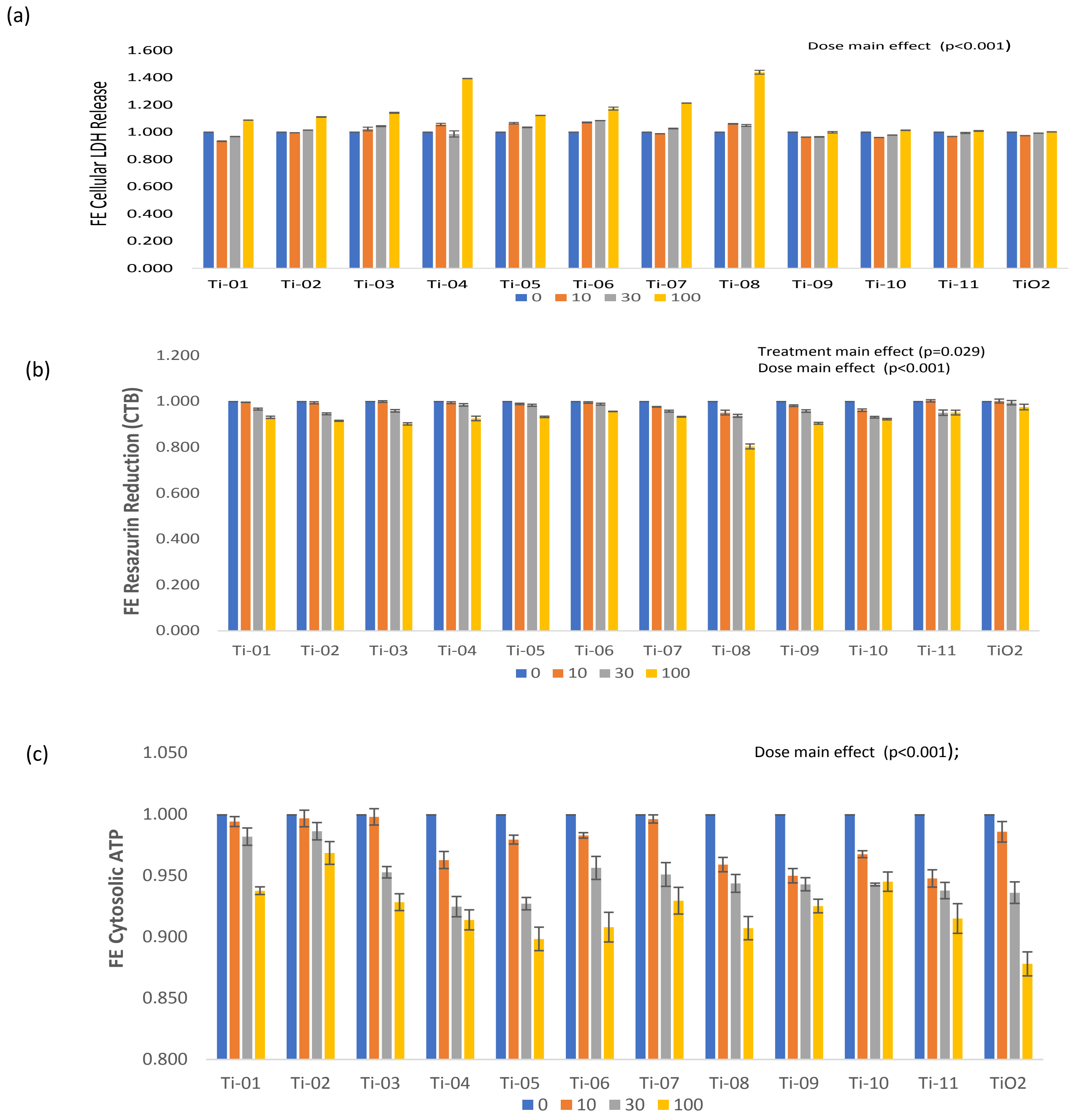

Figure S4. Cytotoxicity analysis in J774A.1 cells exposed to different nanoforms of TiNPs.

Dose-, Treatment-specific cellular cytotoxicity responses ((a) LDH release, (b) CTB reduction (c) ATP levels) of J774A.1 cells exposed to different nanoforms of TiNPs. Triplicate exposure experiments.
